# Detection of Fast Decliner of Diabetic Kidney Disease Using Chiral Amino Acid Profiling: A Pilot Study

**DOI:** 10.1002/cbdv.202403332

**Published:** 2025-02-12

**Authors:** Yosuke Hirakawa, Tomonori Kimura, Shinsuke Sakai, Masayuki Mizui, Masashi Mita, Yoshitaka Isaka, Masaomi Nangaku, Reiko Inagi

**Affiliations:** ^1^ Division of Chronic Kidney Disease Pathophysiology The University of Tokyo Graduate School of Medicine Bunkyo‐ku Japan; ^2^ Division of Nephrology and Endocrinology the University of Tokyo Graduate School of Medicine Bunkyo‐ku Japan; ^3^ Department of Nephrology Osaka University Graduate School of Medicine Suita Japan; ^4^ KAGAMI, Inc. Ibaraki Japan

**Keywords:** diabetic kidney disease | D‐amino acid | D‐serine | fast decliner

## Abstract

Biomarkers for the prediction of diabetic kidney disease are still unsatisfactory. Although D‐amino acids have been shown to reflect kidney conditions, their efficacy in treating diabetic kidney disease (DKD) has not been demonstrated. This study explored the potential role of D‐amino acids as progression markers for DKD, an aspect not addressed previously. We performed comprehensive D‐amino acid measurements and collected the longitudinal estimated glomerular filtration rate (eGFR) data of 135 patients. We defined fast decliners (FDs) as patients exhibiting >10% decline from baseline eGFR per year and compared the D‐amino acid levels of FDs and non‐FDs. Then, we verified that D‐amino acids could predict FDs independent of creatinine levels. In patients with diabetic kidney disease, D‐serine, D‐alanine, and D‐proline were only detected in the blood, while 15 D‐amino acids were detected in the urine. Using supervised orthogonal partial least squares analysis, blood D‐serine and urine D‐amino acid levels were identified as features characterizing diabetic kidney disease. Baseline blood D‐serine levels and ratios did not differ between the FD and non‐FD groups; however, short‐term changes in blood D‐serine levels differed. This study emphasized the significance of D‐serine as a prognostic marker for DKD, an aspect not identified in previous research.

## Graphical abstract

We examined D‐amino acids in diabetic kidney disease (DKD). Among D‐amino acids, blood D‐serine was identified as a biomarker of DKD, and we found that short‐term change in blood D‐serine showed higher value in fast decliners in DKD patients, which meant the prognostic value of blood D‐serine in DKD.

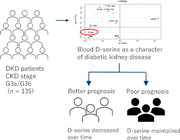

## 1 Introduction

Diabetic kidney disease (DKD) is the leading cause of end‐stage kidney disease worldwide [1]. One of the major problems is the difficulty of clinical prediction. The early detection of patients with poor clinical scenarios enables intensive medical resource allocation. Numerous studies have attempted to identify potential biomarkers. Much effort has been expended on genomic, proteomic, and metabolomic research, and several biomarkers have been identified [2, 3]. However, the newly identified biomarkers still cannot provide sufficient clinical predictions to improve patient prognosis. One reason is that almost all of the pathophysiological effects of biomarkers on DKD remain unknown. The need for new prognostic biomarkers for DKD still exists, preferably biomarkers directly involved in kidney pathophysiology.

Recently, D‐amino acids have been highlighted as biomarkers of kidney disease [4, 5]. D‐amino acids, which are rare enantiomers of the more abundant L‐amino acids, were overlooked until recently [6]. With the advancement of technology, precise measurement of D‐amino acid levels in samples from living organisms is now possible [7]. These analyses have revealed the presence of D‐amino acids in living organisms, including human beings [8]. Notably, the levels of D‐amino acids, especially D‐serine, in the blood increase in patients with kidney disease [8, 9, 10]. This increase is due to the decreased urinary clearance of D‐amino acids [11, 12]. Despite their trace amounts, the key physiological functions of D‐amino acids are also being revealed [13, 14]. Evidence suggests the significance of D‐amino acids in kidney diseases [5, 15, 16], however, their significance in DKD prognosis remains undetermined.

Therefore, we performed a comprehensive amino acid analysis, including a chirality analysis, and examined the prognostic utility of each D‐ and L‐amino acid. We also examined whether intra‐individual fluctuations in amino acids could be a prognostic marker to maximally utilize comprehensive amino acid analysis.

## 2 Results and Discussion

### 2.1 Chiral Amino Acid Analysis of Patients With DKD

**FIGURE 1:**
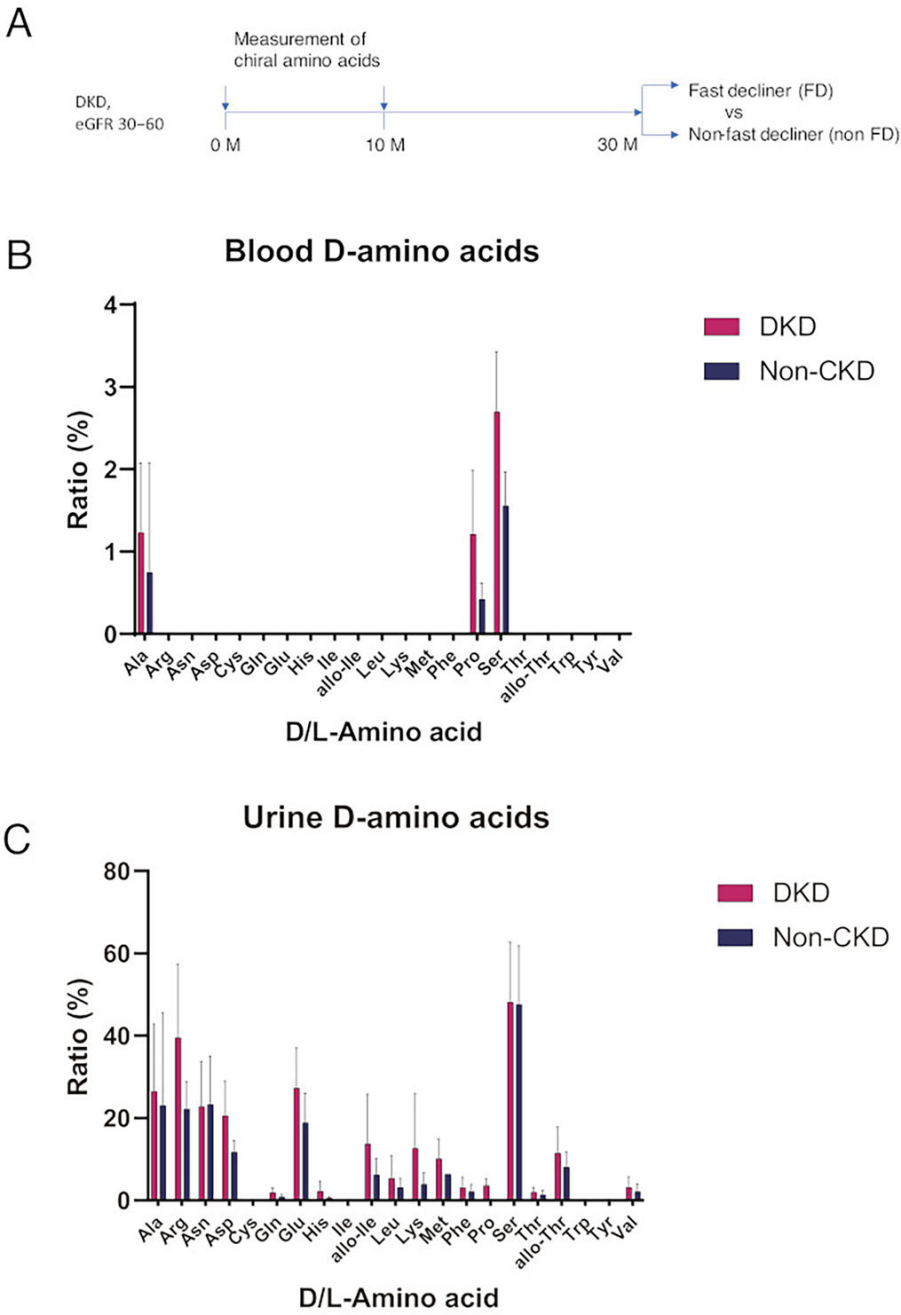
Detection of chiral amino acids in patients with diabetic kidney disease. (A) Schematic of study design. (B and C) D‐amino acid ratios, calculated as the percentage of D‐amino acids to the sum of L‐ and D‐amino acids, in (B) blood and (C) urine. Data, mean ± SD. Abbreviations: CKD, chronic kidney disease; DKD, diabetic kidney disease.

We performed chiral amino acid profiling of patients with DKD to identify metabolites that reflect the rapid decline in glomerular filtration rate. For this analysis, samples of the UT‐DKD cohort, which consisted of DKD patients with estimated glomerular filtration rate (eGFR) ranging from 30 to 60 mL/min/1.73 m^2^, were used (Figure 1A). The baseline characteristics of the patients are shown in Table 1. A total of 135 patients with DKD were included in this study. We also utilized reference data on chiral amino acid metabolomics from a cross‐sectional cohort that included healthy volunteers (*n* = 15, non‐chronic kidney disease [CKD] group) [9]. Patients with DKD were older and had a higher body mass index. Although age and BMI could act as confounding factors, these differences cannot be excluded because DKD is more prevalent in older and obese individuals.

**TABLE 1:** Background demographics of participants.

Characteristics	DKD N = 135	Non‐CKD N = 15
Male	107 (79%)	12 (80%)
Age (year)	72 [66–76]	44 [39–50]
Body mass index (kg/m^2^)	25.4 [23.3–27.3]	22.6 [21.1–25.7]
Serum Creatinine (mg/dL)	1.16 [0.99–1.31]	0.75 [0.68–0.83]
eGFR (mL/min/1.73m^2^)	47.8 [41.6–53.4]	87.3 [79.3–93.5]
SBP (mmHg)	141 [130–154]	125 [120–140]
DBP (mmHg)	72 [64–79]	89 [81–94]
HbA1c (%)	6.8 [6.5–7.2]	NM

Abbreviations: CKD, chronic kidney disease; DBP, diastolic blood pressure; DKD, diabetic kidney disease; eGFR, estimated glomerular filtration rate; HbA1c, hemoglobin A1c; NM, not measured; SBP, systolic blood pressure. Reference data for non‐CKD were obtained from a secondary analysis of a previous report [9].

Chiral amino acid analysis of patients was performed using serum and urine samples. D‐Amino acids detected in more than 30% of the samples are shown in Figure 1B,C, and Figure S1. Three major D‐amino acids–D‐alanine, D‐serine, and D‐proline–were detected in the blood of both the DKD group and the non‐chronic kidney disease (non‐CKD) group. The concentrations of these D‐amino acids exhibited substantial inter‐individual variability, which limited the statistical power of direct comparisons of D‐amino acid ratios between the DKD and non‐CKD groups. The ratios of D‐amino acids were trace and only around 1%–3%. D‐Amino acids were much more abundant in urine. Fifteen types of D‐amino acids were detected in the urine, with ratios ranging up to 50%.

### 2.2 Blood D‐Serine Is Characteristic of DKD

**FIGURE 2:**
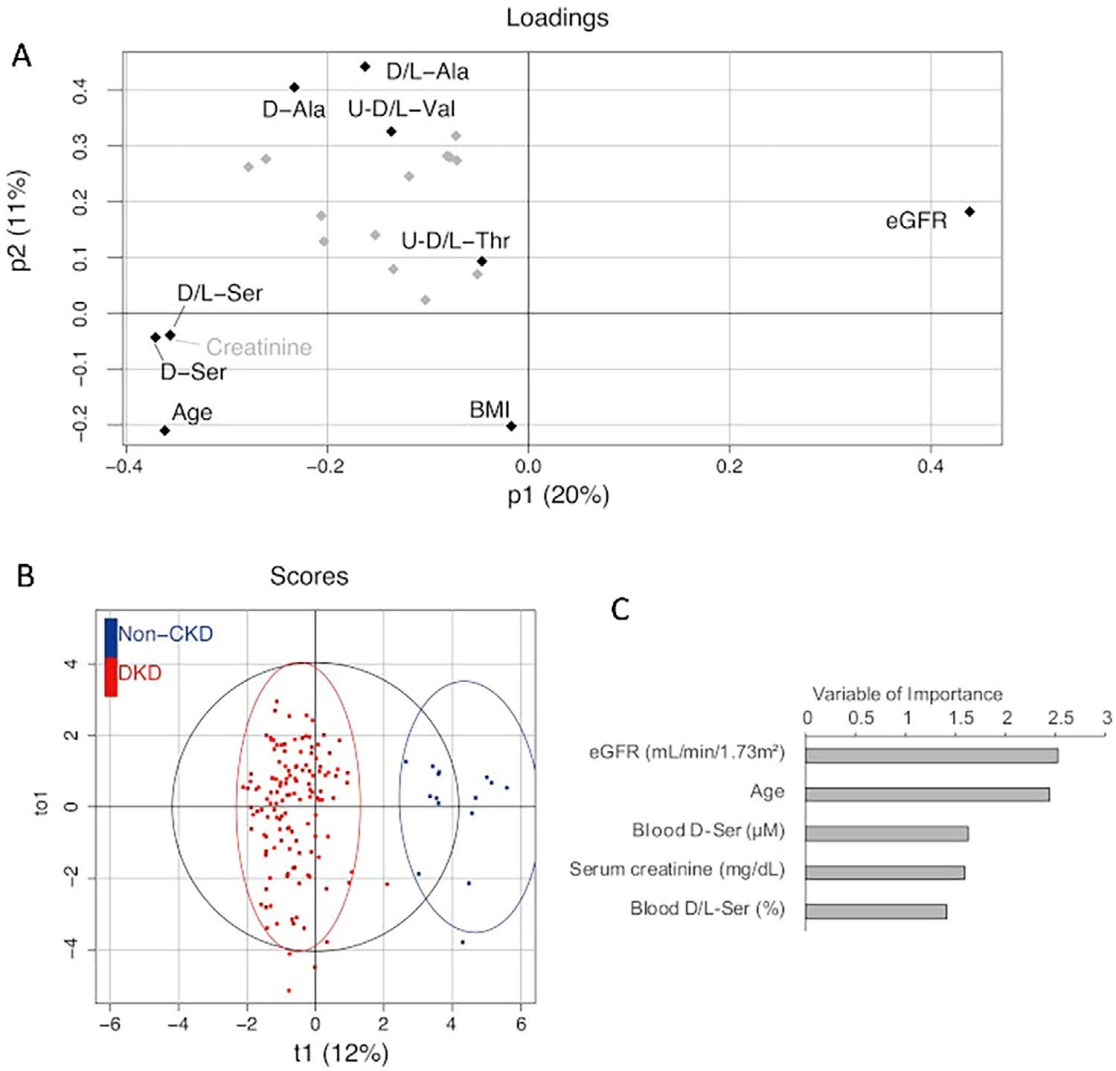
Blood D‐serine is characteristic of DKD. (A) Orthogonal partial least squares (OPLS) analysis of D‐amino acids on DKD versus non‐CKD. Models were developed using blood levels and ratios of D‐amino acids and clinical parameters. (B) Score plot of OPLS colored according to DKD and non‐CKD status. The circle represents a 95% confidence interval. (C) Key variables important for discriminating DKD. Abbreviations: CKD, chronic kidney disease; DKD, diabetic kidney disease.

To explore the metabolites that are characteristic of DKD, we performed an OPLS analysis of the discriminable capacity of chiral amino acids between DKD and non‐CKD participants (Figure 2A). These models were developed in combination with clinical parameters and chiral amino acids. The levels and ratios of blood D‐amino acids were included, whereas only the ratios of urine D‐amino acids were included because raw urine data are affected by concentration. The loading plot showed that eGFR had a strong discriminatory capacity, whereas blood D‐serine, blood D‐serine ratio, and serum creatinine were present on the opposite sides (Figure 2B). The score plot showed good discrimination of this model. As key variables in this model, eGFR was selected, followed by age, blood D‐serine level, serum creatinine level, and blood D‐serine ratio (Figure 2C). Blood D‐serine has been identified as a characteristic metabolite of DKD.

### 2.3 Increased Level and Ratio of Blood D‐Serine in DKD

**FIGURE 3:**
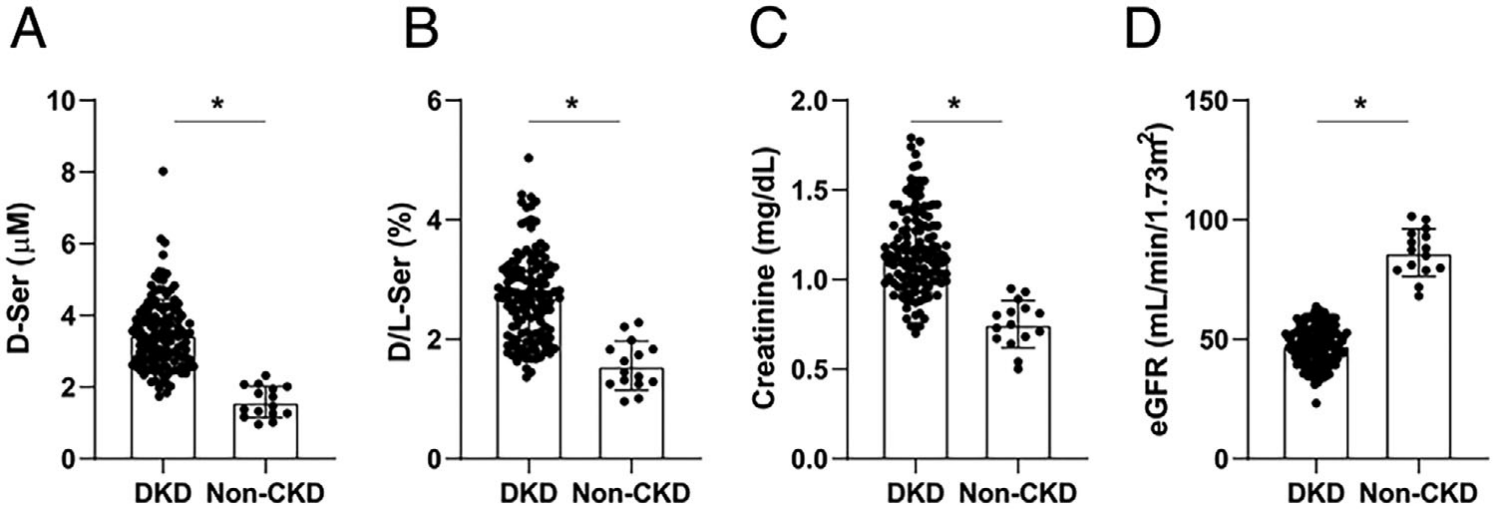
Increased level and ratio of blood D‐serine in DKD. (A) D‐serine level, (B) D‐serine ratio, (C) blood creatinine, and (D) estimated glomerular filtration rate (eGFR) of each group. *: *p* < 0.05. Abbreviations: CKD, chronic kidney disease; DKD, diabetic kidney disease.

We analyzed baseline data for D‐serine levels (Figure 3A). Blood D‐serine levels are higher in patients with DKD than in non‐CKD. Similarly, the ratio of D‐serine in the blood was higher in patients with DKD than in non‐CKD (Figure 3B). As expected, serum creatinine levels were higher and the eGFR was lower in patients with DKD than in those without CKD (Figure 3C,D). The D‐serine profile was similar to that of serum creatinine, suggesting that D‐serine also reflects kidney function in DKD.

### 2.4 Increase in Blood D‐Serine Level Is a Predictor of the Decline in Estimated Glomerular Filtration Rate

Finally, we examined whether the levels and ratios of D‐serine in the blood reflected the prognosis of kidney function. Using the decline in creatinine‐based eGFR as a reference, the D‐serine levels and ratios were examined between the FD and non‐FD groups. FD was defined as a 10% decline from the baseline eGFR per year, confirming a significant decline in kidney function. In the baseline analysis, there were no differences in the levels and ratios of D‐serine in the blood between the FD and non‐FD groups, whereas the FD group had slightly worse serum creatinine levels and eGFR (Figure S2). This was a reflection of analyzing the creatinine‐based outcome; naturally, creatinine may be more sensitive in detecting differences.

**FIGURE 4:**
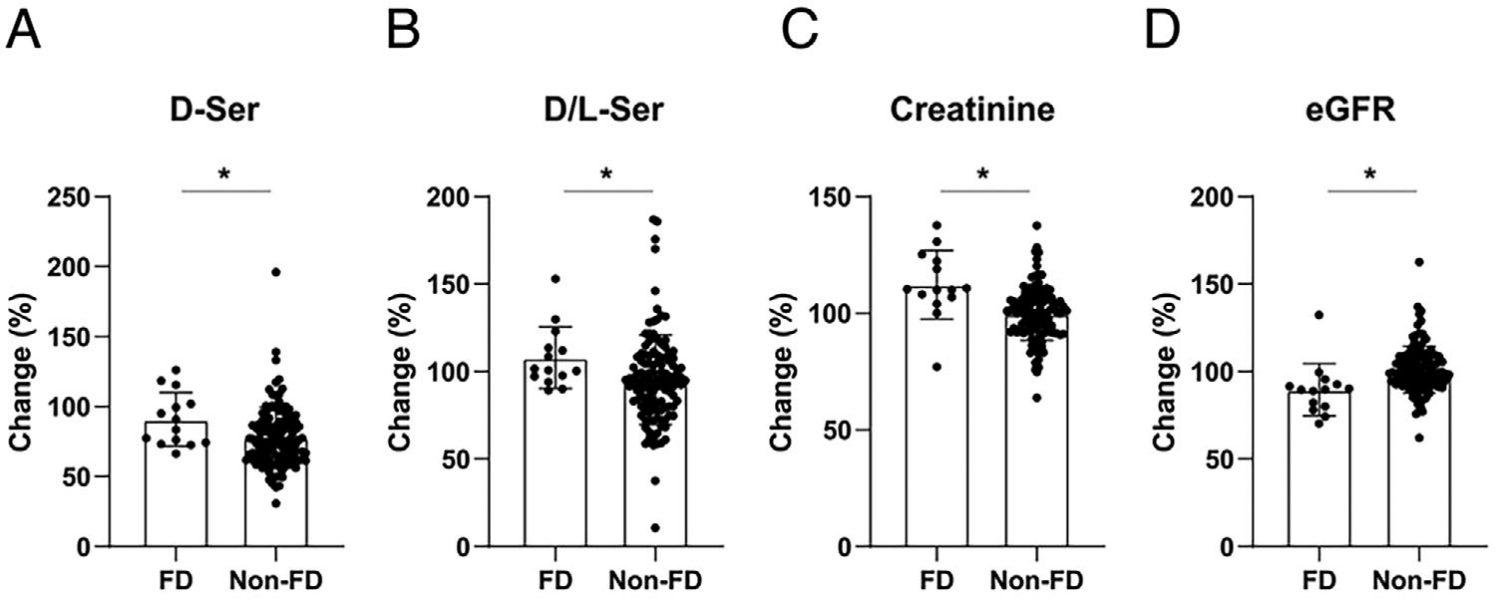
Increase in blood D‐serine level precedes the decline in estimated glomerular filtration rate. Changes were calculated by dividing the second values by the first. (A) D‐serine level, (B) D‐serine ratio, (C) blood creatinine, and (D) estimated glomerular filtration rate (eGFR) of each group. *: *p* < 0.05. Abbreviation: FD, fast decliners.

After observing the similarity in baseline levels, we wondered whether changes in D‐serine levels would predict the worsening of kidney function. Therefore, we calculated short‐term changes in D‐serine levels by dividing the second value by the first value as shown below (Figure 4A).

D−Serchange=2ndD−Ser/1stD−ser

D/L−Serchange=2ndD/L−Ser/1stD/L−ser

In the entire cohort, the D‐serine level was lower in the second sample than in the first. In the grouped analysis, D‐serine levels were significantly lower in the non‐FD group than in the FD group. Similarly, the D‐serine ratio decreased in non‐FD, while it did not in FD, although the trend was marginally significant (Figure 4B). These trends were largely similar to those of serum creatinine levels and eGFR; approximately half of the non‐FD patients showed a decline in serum creatinine levels and an increase in eGFR, but the FD group did not (Figure 4C, D). Increased blood D‐serine levels preceded a decline in the eGFR.

## 3 Discussion

In this study, we examined whether amino acid enantiomers can be used as progression markers. We detected three major D‐amino acids, D‐alanine, D‐serine, and D‐proline in the blood and 15 D‐amino acids in the urine of patients with diabetic kidney disease and healthy subjects. Among these D‐amino acids, blood D‐serine was identified as a key feature of DKD. These findings are consistent with those of previous studies [8, 9, 16]. We found that changes in blood D‐serine concentration and the D/L‐serine ratio preceded a decline in kidney function. This point, that short‐term changes in blood D‐serine have additional utility in predicting prognosis, has not been shown before, since data on longitudinal D‐amino acid concentrations were limited.

The dynamics of D‐amino acids have been increasingly elucidated in recent years. Unlike other D‐amino acids, D‐serine can be endogenously produced in the mammalian brain through the activity of the enzyme serine racemase [17]. In contrast, other D‐amino acids cannot be synthesized by mammals and are entirely dependent on dietary intake. Orally ingested D‐amino acids, such as D‐alanine, transiently elevate plasma levels, which subsequently return to near basal levels, indicating rapid clearance [18]. Since the clearance of D‐amino acids is primarily mediated by glomerular filtration, they have been proposed as potential functional markers of renal function [11, 19]. Among the D‐amino acids, D‐serine has been the most extensively studied in relation to kidney disease.

D‐serine is a disease marker of CKD and has also been reported to be a diagnostic marker of DKD. Fifty‐nine patients with diabetic kidney disease were distinguished from 247 patients with primary glomerulonephritis by comparing the urinary fractional excretion of D‐serine [16]. This study evaluated the diagnostic utility of blood and urine D‐serine levels but did not assess their prognostic value following a diagnosis of DKD. A study that included 108 patients with CKD showed that high baseline blood D‐serine levels were associated with poor kidney outcomes. In this study, blood D‐serine levels can be considered a prognostic marker, but the participants of this study had various background diseases, and only 30% of the participants had diabetic kidney disease [8]. As described above, diabetic patients showed unique D‐serine dynamics; therefore, analysis of the diabetic cohort must highlight its specific prognostic utility in DKD. In our study, D‐serine was one of the characteristic features of patients with diabetic kidney disease, which is consistent with previous reports. The baseline levels and ratios of D‐serine did not differ between the FD and non‐FD groups. This inconsistency with a previous report can be explained in terms of the cause of CKD; that is, all participants in this study had DKD. In contrast, a short‐term increase in blood D‐serine levels had an independent predictive effect on eGFR decline. These results indicate that inter‐individual comparison of blood D‐serine levels cannot yield a precise evaluation of kidney conditions because of the wide inter‐individual variation in blood D‐serine levels, even with a similar eGFR. However, intraindividual fluctuations in D‐serine levels are worthy of attention and could be a prognostic marker other than creatinine or eGFR.

There is a growing need for novel biomarkers of DKD, particularly prognostic biomarkers. Traditional prognostic factors for DKD include age, blood pressure, and albuminuria [20], but prognosis prediction models based on clinical factors alone have limited utility. Therefore, novel biomarkers have been identified. Plasma tumor necrosis factor receptor (TNFR)‐1, TNFR‐2, and kidney injury molecule‐1 have been identified as prognostic markers, and numerous biomarkers have been identified in response to developments in genomics, transcriptomics, proteomics, and metabolomics [21]. However, biomarkers have rarely been implemented in patient care or used in drug development. One reason why the identification of biomarkers cannot lead to subsequent development is the lack of clarity regarding their pathophysiological functions. From this perspective, the advantage of utilizing D‐serine is that it has a protective effect against kidney disease. Oral administration of D‐serine in mice resulted in increased blood levels of D‐serine and mitigated kidney damage following ischemia‐reperfusion injury [22]. In another report, D‐serine supplementation promoted the remodeling of the mouse kidney after unilateral kidney resection [14]. Considering the unique dynamics of D‐serine in patients with diabetes, D‐serine dysregulation may play a role in the progression of diabetic kidney disease.

Limitations of this study must be addressed. First, it was a single‐center pilot study. To confirm the predictive effect of D‐serine on the progression of diabetic kidney disease, multicenter studies with a larger number of patients are needed. Second, the characteristics of DKD may have been confounded by factors such as advanced age and obesity. Third, owing to the limited number of patients, we could not perform multiple regression analyses that included all conventional risk factors for diabetic kidney disease progression. Fourth, we showed only a correlation between short‐term D‐serine accumulation and DKD progression and not the pathological role of D‐serine in DKD progression.

## 4 Conclusion

We identified D‐serine as a characteristic feature of patients with DKD. The concentration of D‐serine is dependent on kidney function, whereas a short‐term increase in D‐serine is an independent predictor of early decline in DKD. Considering that D‐serine has pathophysiological effects on kidney disease, D‐serine fluctuation deserves future vigorous examination as a predictor of DKD.

## 5 Methods

### 5.1 Study Design and Approval

This study was approved by the ethics committees of the University of Tokyo Graduate School of Medicine (10660) and Osaka University Graduate School of Medicine (23258) and conducted in compliance with the Declaration of Helsinki. Details of the UT‐DKD cohort have been described elsewhere [3, 23]. The UT‐DKD cohort consisted of patients with CKD and G3a/G3b DKD without any obvious kidney diseases other than DKD. A total of 150 patients were recruited between January 2015 and September 2016. Plasma and urine samples were collected at baseline and follow‐up visits. Continuous eGFR data were collected until the final eGFR value was reached 30 months after the baseline visit. A total of 135 patients completed their final visit. Fast decliners were defined as patients exhibiting >10% decline in eGFR per year from baseline eGFR, which corresponds to a surrogate endpoint as a %GFR change of less than −30% over 2 or 3 years [24]. The annual rate of eGFR decline was calculated every 10 months using the least‐squares method. The D‐amino acid data of healthy volunteers were obtained from a secondary analysis of a previous report [9]. Written informed consent was obtained from all of the participants.

#### 5.1.1 Measurement of Chiral Amino Acids Using Two‐Dimensional High‐Performance Liquid Chromatography

Chiral amino acids were quantified using two‐dimensional high‐performance liquid chromatography (2D‐HPLC) as described previously [7, 8]. The samples were prepared as follows. Twentyfold volumes of methanol were added to the sample and an aliquot (10 µL of the supernatant obtained from the methanol homogenate) was placed in a brown tube. After drying the solution under reduced pressure, 20 µL of 200 mM sodium borate buffer (pH 8.0) and 5 µL of fluorescence labeling reagent (40 mM 4‐fluoro‐7‐nitro‐2,1,3‐benzoxadiazole in anhydrous acetonitrile) were added and heated at 60°C for 2 min. An aqueous solution of 0.1% (v/v) trifluoroacetic acid (75 µL) was added, and 2 µL of the reaction mixture was subjected to 2D‐HPLC.

In the 2D‐HPLC platform, the fluorescence‐labeled amino acids were separated using a reversed‐phase column (Singularity RP column, 50 mm × 1.0 mm i.d., 3‐µm particle size; KAGAMI INC., Osaka, Japan), with the gradient elution using aqueous mobile phases containing acetonitrile and formic acid. The concentration of acetonitrile and formic acid varied from 1% to 40% and from 0.01% to 0.5%, respectively, depending on the target amino acid. For example, 1% acetonitrile and 0.03% formic acid was used for D/L‐serine. To determine D‐ and L‐amino acids separately, the fractions of amino acids were automatically collected using a multi‐loop valve and transferred to the enantioselective column (Singularity CSP‐001S, 75 mm × 1.5 mm i.d., 5‐µm particle size; KAGAMI INC.). Additionally, D‐ and L‐amino acids were separated in the second dimension using an enantioselective column. The mobile phase was a mixed solution of 0%–90% methanol (90% for D/L‐serine), 10%–100% acetonitrile (10% for D/L‐serine), and 0.005%–1% formic acid (0.04% for D/L‐serine) were used depending on the target amino acid. Fluorescence was detected at 530 nm with excitation at 470 nm. The quantitative 2D‐HPLC system was calibrated before and after each test, demonstrating a robust precision with a standard deviation of 1.68%. The accuracy was measured at 102.72% across five batches tested on different days.

#### 5.1.2 Statistical Analysis

Continuous variables were presented as medians and ranges or means and standard errors. Categorical variables are presented as ratios (%) and counts. The discriminable distributions of variables based on predictive variables were analyzed using supervised orthogonal partial least squares analysis [12]. The D‐amino acid ratio was defined as the ratio of D‐amino acids to the sum of L‐ and D‐amino acids. Metabolites detected in more than 30% of the samples were minimally imputed and analyzed. Continuous variables were compared between groups using the Wilcoxon rank‐sum test. Statistical significance was defined as *p* < 0.05. Statistical analyses were performed using the PRISM, STATA, and R software.

## Conflicts of Interest

The authors declare no conflicts of interest.

## Supporting information



Supporting Information

## Data Availability

The data that support the findings of this study are available from the corresponding author upon reasonable request.
