# Supplementary material for: Detection of Fast Decliner of Diabetic Kidney Disease Using Chiral Amino Acid Profiling: A Pilot Study
Source: Chem Biodivers. 2025 Feb 12;22(6):e202403332. doi: 10.1002/cbdv.202403332 (PMC12168192; doi:10.1002/cbdv.202403332)
Supplement: Supplementary file 1 — Supporting Information [file CBDV-22-e202403332-s001.pdf]

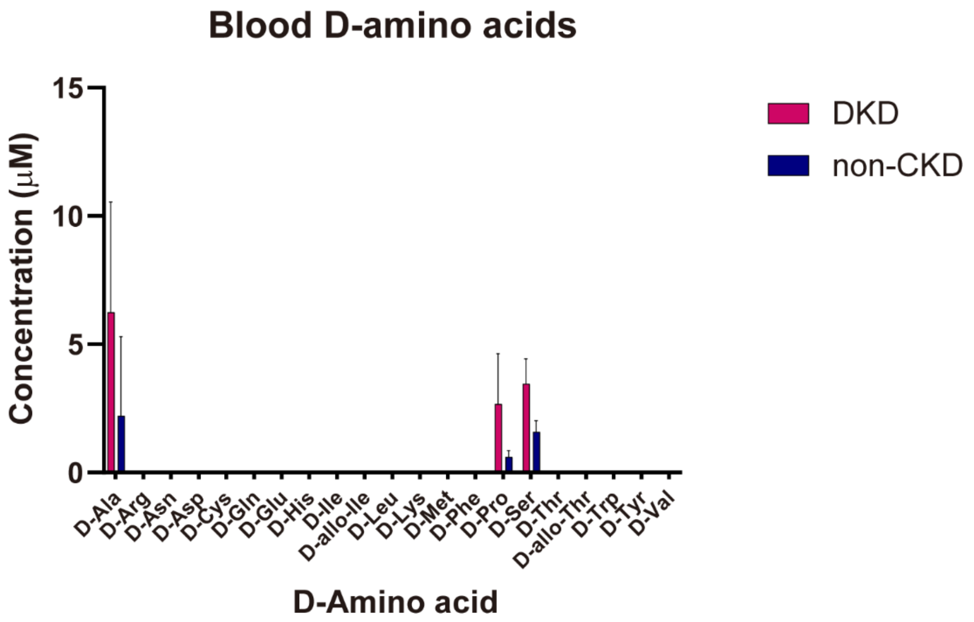

Supporting Figure 1. Detection of chiral amino acids in patients with diabetic kidney disease. D-Amino acid levels in the blood. Data, mean ± SD. Abbreviations: CKD, chronic kidney disease; DKD, diabetic kidney disease.

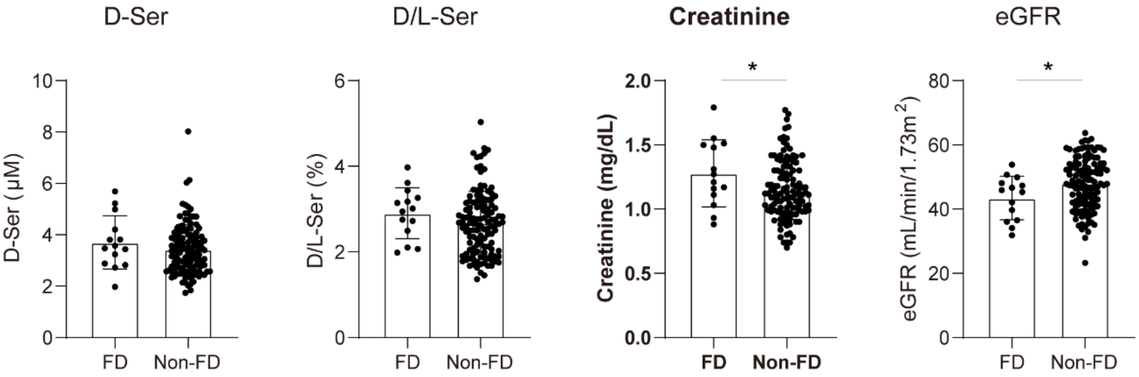

Supporting Figure 2. Baseline level and ratio of blood D-serine in DKD. (A) D-serine level, (B) D-serine ratio, (C) blood creatinine, and (D) eGFR in each group. Data, mean ± SD. *P < 0.05. Abbreviation: FD, fast decliners.
